# Real Impact of Novel Immunotherapy Drugs in Cancer. The Experience of 10 Last Years

**DOI:** 10.3390/toxins13020149

**Published:** 2021-02-15

**Authors:** Andreas Koulouris, Christos Tsagkaris, Michail Nikolaou

**Affiliations:** 1 Department of Medical Oncology University General Hospital of Heraklion, University of Crete, 71003 Heraklion, Greece; andreas.koulouris@gmail.com; 2 Faculty of Medicine, University of Crete, 71003 Heraklion, Greece; chriss20x@gmail.com; 3 1st Oncology Department, “Saint Savas” Anticancer—Oncology Hospital, 11522 Athens, Greece

**Keywords:** immunotherapy, checkpoint inhibitors, cancer treatment, toxicity, PD-1, PD-L1, CTLA-4

## Abstract

Intense research on immunotherapy has been conducted during recent years. As advances in the field have started changing the landscape of cancer therapy, it is necessary to assess the impact of immunotherapeutic modalities in the treatment of various cancers. Ten years ago, in 2011, ipilimumab was the first of the newest immunotherapeutic drugs against cancer to be approved by the FDA. Then several drugs followed and formed a therapeutic arsenal to fight cancer. Initial studies were performed on metastatic patients, but there are currently several studies in patients with potentially curable cancers. All these developments have created a new environment for oncology which we will present in this article. This review examines the current evidence related to the impact of immunotherapy on various cancers and discusses its potential clinical and research implications, including its effectiveness in comparison to other treatment modalities (chemotherapy, radiotherapy), its toxicity and prospective research opportunities. While constant updates and further research is critical to understand the impact of immunotherapy in cancer therapy, not only does it seem to be important to assess the current state of knowledge highlighting the success but also to determine the challenging aspects of cancer immunotherapy.

## 1. Introduction

Immunologic manipulations to control tumor growth can essentially be either passive or active. The difference between active immunotherapy and passive immunotherapy is related to their impact on the immune system of the patient. Active immunotherapy aims to produce a durable immune response on the grounds of induced immunological memory. The concept of active immunotherapy simulates a physiological immune response [1].

Agents commonly administered in the context of active immunotherapy consist of interferons and interleukins, as well as microbial strains. The bacillus Calmette Guerin (BCG), the microorganism causing tuberculosis, is a typical example of active immunotherapy administered in the context of non-muscle invasive urinary bladder cancer. The immune response induced by BCG has been proven to lead this type of cancer to regression [2,3].

Passive immunotherapy substitutes the roles of the human immune system without requiring an active response of the immune system to cancer cells. Passive immunotherapy consists of administering immunologic reagents, including serum, cells, or cell products with antitumor activity, to a tumor-bearing patient. Monoclonal antibodies (mAb’s) represent the largest class of commercially available passive cancer immunotherapies to date and are directed to a single cellular target [4].

Anticancer immunotherapeutic regiments that have received approval by regulatory authorities all over the globe so far include dendritic cell-based immunotherapies (Sipuleucel-T), immunostimulatory cytokines (IFN-α2a, IL-2 and IL-12), immunomodulatory mAb’s (ipilimumab, nivolumab, pembrolizumab, etc.), oncolytic viruses (vitespen), pattern recognition receptor (PRR) agonists (BCG, Picibanil, Imiquimod) [3]. Anticancer immunotherapies whose approval is still pending include adoptive cell transfer, DNA-based vaccines and inhibitors of immunosuppressive metabolism. Striking examples of adoptive cell transfer are: (a) the adoptive immunotherapy with cytotoxic T-lymphocytes which are specific for malignant cells or particular antigens and (b) CAR T-cells that bear chimeric antigen receptors (CARs) against cancer cells, which reveal promising results in the treatment of lymphomas. 

During the past decades, anticancer immunotherapy has evolved from a promising research concept to an incremental part of clinical oncology. Not only has a wide variety of immunotherapeutic agents been licensed by the US Food and Drug Administration (FDA) and the European Medicines Agency (EMA), which have already been available for use in cancer patients, but also many other agents are under investigation. In most cases, immunotherapy is administered as an addition to conventional therapeutic means. Moreover, there is a growing tendency of developing existing or novel immunotherapeutic agents as standalone therapeutic interventions [2]. To the present, immune checkpoint inhibitors (ICIs) have gained widespread adoption and they have already been the major immunotherapeutic approach; therefore, ICIs will be the most thoroughly described therapeutic approach in the following results. However, the adoption of ICIs and immunotherapeutics, in general, has been forged through a long journey of doubt, promise and experimentation. Revisiting the milestones of the evolution of immunotherapy for cancer, can boost our current understanding and unravel potential aspects of innovation on a problem-solving basis (Table 1).

## 2. The Evolution of Immunotherapy for Cancer

The inception of anticancer immunotherapy can be traced back to Hippocrates as a concept although we should seek its whereabouts in the work of William Coley [5]. The initial concept includes the understanding of the fact that the immune system, a “natural force” can be either activated or mimicked consists of the core of anticancer immunotherapy. It would take more than 20 centuries though, for the understanding of medicine to abandon the humoral pathology, adopt the cellular paradigm and dare to explore the immune system. The first hint of the potential contribution of immunological factors to tumor regression comes from the early 1900s when Dr. William Coley, a surgeon in New York, observed the regression of a round cell sarcoma following a bacterial infection on the site of the operation [6,7,8]. Judging from that particular case, Coley hypothesized that an artificially produced infection over a cancerous lesion could be a novel treatment approach. This experience motivated Coley to experiment more with the technique of inoculation so that he can induce infection and a subsequent immune response promptly. Later on, Coley developed a technique of cancerous lesions contamination based on heat-killed bacteria, a treatment that became known as “Coley toxins” [5].

Despite the spectacular effect of this primitive immunotherapy, its etiological basis was not understood. Its effectiveness proved to be sporadic and when radiotherapy appeared, Coley toxins were abandoned for long. The concept of Coley toxins remained intriguing sparkling relevant basic research attempts. Between 1930 and 1945, several animal studies with bacteria contaminating various tumors were conducted. The principal findings were that not only did bacteria cause tumor necrosis, but also serum from endotoxin-treated tumors could be reintroduced to tumors [8].

The fact that the serum contributed to tumor necrosis, led researchers to conclude that it contained a “tumor necrotizing factor”. In the next years, investigators comprehended that the destruction was invoked by host cells as a response to bacterial endotoxins and not by the endotoxin itself. Subsequently, the term was modified to tumor necrosis factor (TNF). TNF failed as a systemic treatment because of serious side effects including fever, rigor, headache, hypotension and pulmonary edema [8,9]. 

Soon after, researchers at John Hopkins University observed a lower prevalence of cancer among patients who have recovered from tuberculosis. This observation led to the use of BCG as anticancer immunotherapy. However, clinical implementation was halted for about thirty years due to an unfortunate administration of a contaminated vaccine to numerous newborns in Germany, also known as the Lubeck disaster [10].

A decisive turn in the history of anticancer immunotherapy was observed in the early 1970s, when the discovery of IL-2 sparked hypotheses that led to promising results. From 1990 onwards intense research is conducted including real-world data from clinical trials and every day clinical practice [11].

The future history of anticancer immunotherapy is expected to be more fascinating taking into consideration the potential that novelties such as artificial intelligence, machine learning, the internet of things and quantum computing tend to play a more active role in healthcare. Accelerated screening and identification of therapeutic targets, immunotherapeutic agents’ repurposing and clinical trial data analysis are only some of the features that have the potential to speed up the evolution of cancer immunotherapy unraveling new therapeutic paths [12].

In the meantime, the key concept of the evolution of cancer immunotherapy has yet to be deeply understood. A concept can be discovered by mistake or through an unfortunate incident. Even though its development can be halted, it might still be worthy of being revisited when knowledge progresses to reveal previously hidden insights.

Overall, the accumulating wealth of knowledge concerning anticancer immunotherapy needs to be promptly assessed and assimilated in clinical practice and medical/postgraduate education. Hence, the purpose of this article is to review the status of immunotherapy impact in new anti-cancer treatments.

## 3. Methods 

The current evidence about immunotherapy impact on new anti-cancer treatments is reviewed in this article. Particularly, preclinical and clinical data from phase I/II clinical trials, which evaluate the efficacy and toxicity of immunotherapeutic agents for patients with cancer, are summarized based on PubMed/MEDLINE search and relevant articles, which have been presented at international conferences. In addition, future perspectives about the emerging role of immunotherapy regarding variant types of cancer, such as small cell lung cancer (SCLC) are highlighted in light of potentially useful biomarkers and health policy. 

We searched Pubmed and Google Scholar with the following strategy: {[prospective (Title/Abstract) OR cohort (Title/Abstract) OR follow-up (Title/Abstract) OR review (Title/Abstract) OR longitudinal (Title/Abstract) OR meta-analysis (Title/Abstract) OR systematic review (Title/Abstract)] AND [immunotherapy (Title/Abstract) OR cancer (Title/Abstract)] AND [cancer treatment OR melanoma OR colon cancer OR urothelial cancer OR lung cancer OR breast cancer (MeSH Terms)]}, up until September 2020. This search strategy aims to identify: (1) Clinical trials involving immunotherapy; (2) Other original studies or metanalyses related to the use of immunotherapy. Original, peer-reviewed studies in English were included. 

## 4. Results

**Figure 1:**
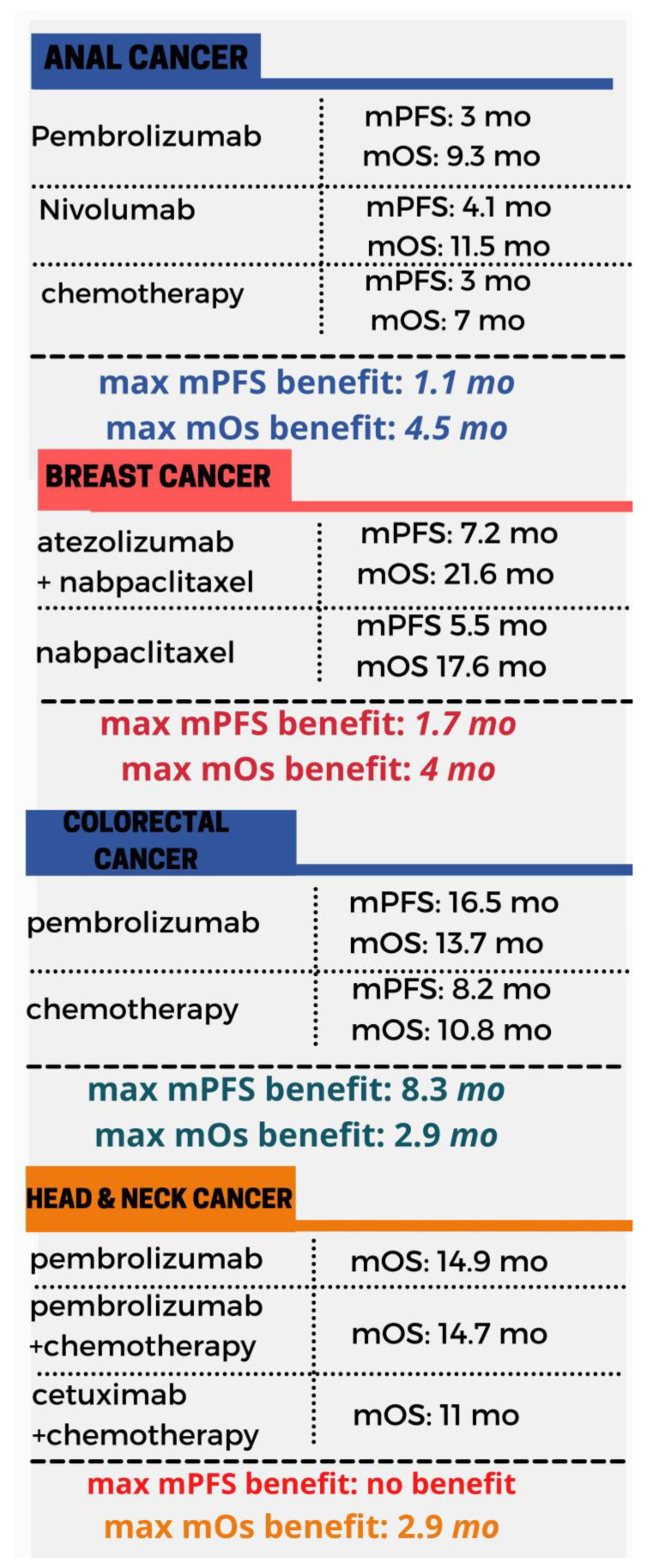
An overview of the mPFS and mOS in the context of anal [15], breast [18,19], colorectal [21] and head and neck cancer [28,29]. mPFS, median progression-free survival, mOS, median overall survival.

**Figure 2:**
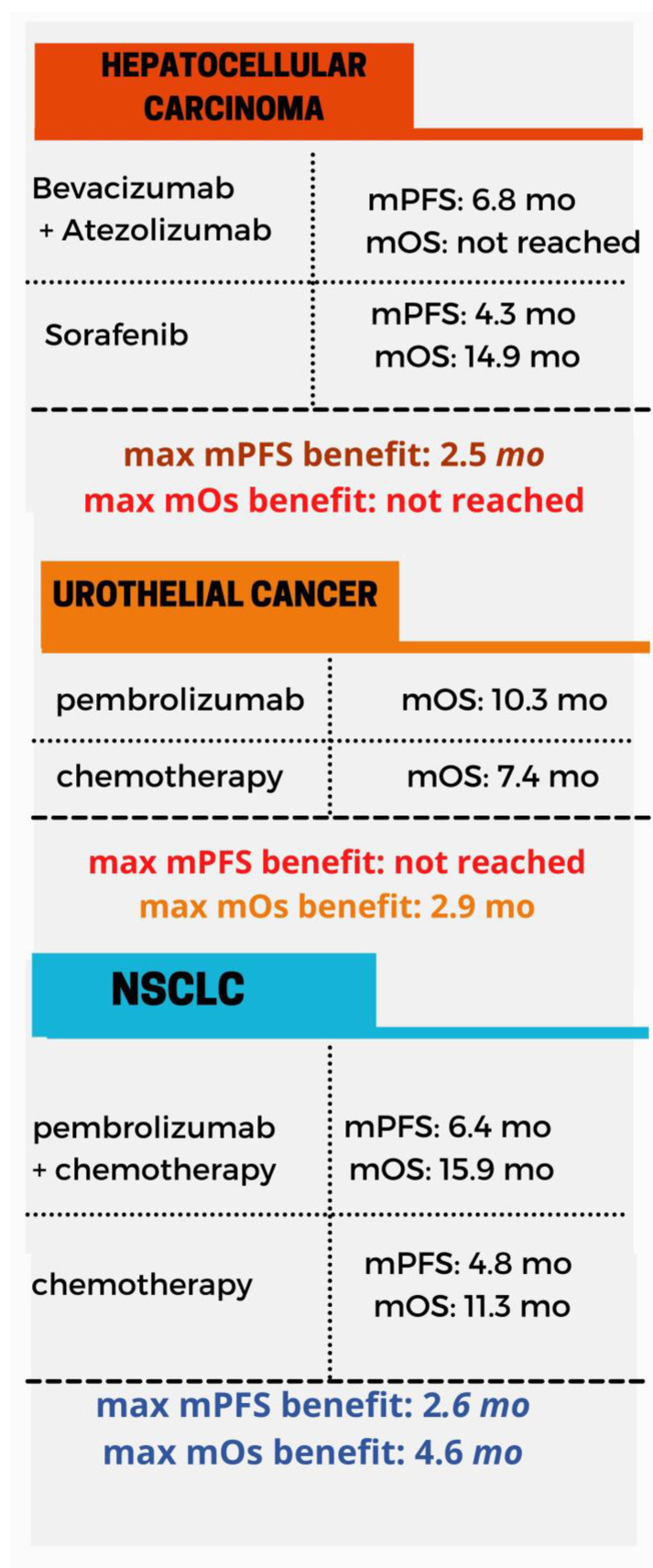
An overview of the mPFS and mOS in the context of hepatocellular carcinoma [37,38,39], urothelial [87,89,90] and non-small cells lung cancer [49,50]. mPFS, median progression-free survival, mOS, median overall survival.

**Figure 3:**
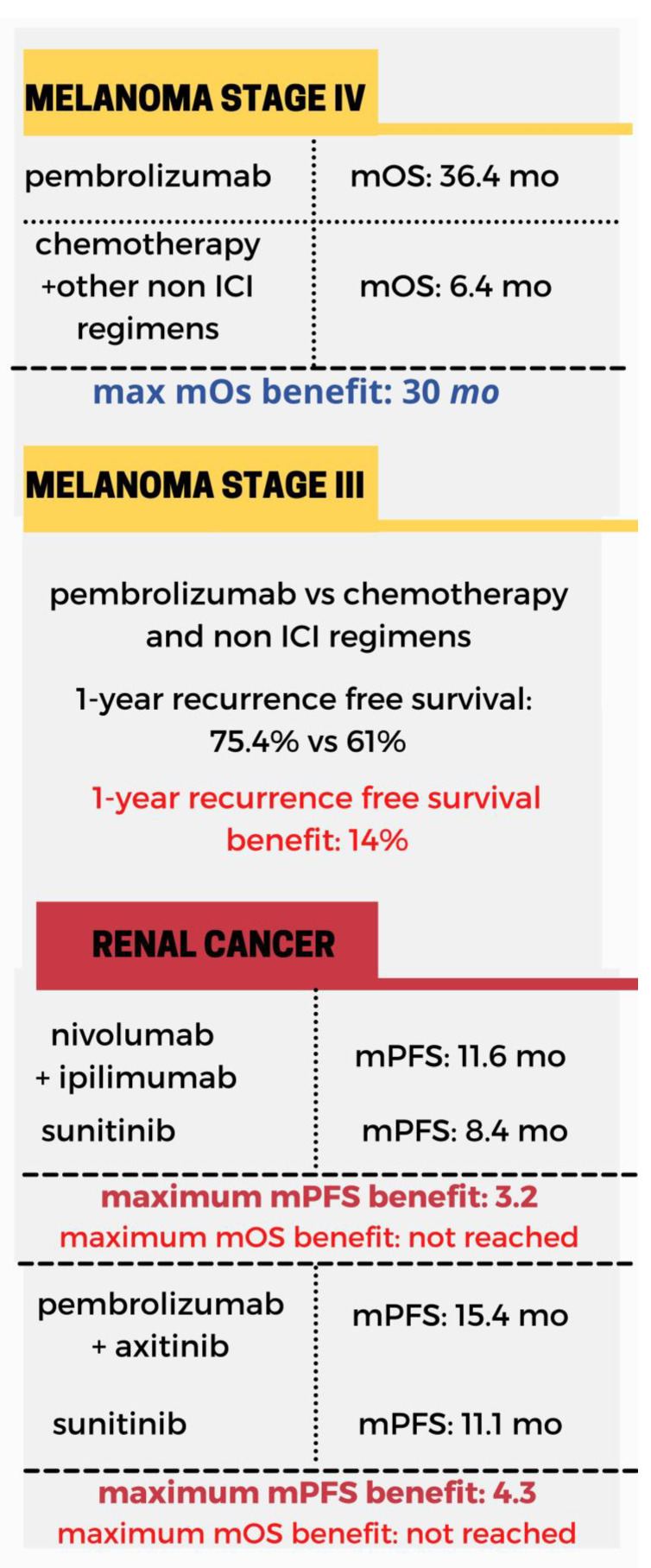
An overview of the mPFS and mOS in the context of melanoma [72] and renal cancer [81,83] mPFS, median progression-free survival, mOS, median overall survival.

Immunotherapy has been deployed in the context of anal, breast, colorectal and head and neck cancer (Figure 1), hepatocellular carcinoma (HCC), urothelial, non-small-cell lung carcinoma (NSCLC) (Figure 2) melanoma and renal cancer (Figure 3). In these malignancies there is comparative evidence between immunotherapy and the current state of treatment in terms of mean progression-free survival (mPFS) and mean overall survival (mOS). Applications of immunotherapy in other cancers including urothelial cancer, lymphoma, prostate cancer, gynecological malignancies and soft tissue sarcomas are reviewed in the context of ongoing clinical trials.

### 4.1. Anal Cancer

Immunotherapy has been demonstrated to be beneficial to patients with refractory metastatic anal squamous cell carcinoma (SCC) with progression disease (PD) after chemotherapy. The anti-PD1 moAbs, Pembrolizumab and nivolumab, as well as, the PD-L1 moAb, durvalumab, have been studied in the metastatic disease showing promising results in terms of PFS and OS [13]. Specifically, according to the phase Ib KEYNOTE-028 study, pembrolizumab has been tested in PD-L1 positive patients achieving overall response rate (ORR) 17%, whereas its median PFS period was 3 months and its median OS was 9.3 months in comparison with an OS of 7 months when chemotherapy with mitomycin C and 5FU was tested as second line of treatment [95% CI 2.2–11.8] [14,15]. Diarrhea, fatigue and nausea were included in the most common treatment-related side effects [14,15]. Similar results have been documented in the phase 2 NCI9673 study of nivolumab. Its median PFS was estimated at 4.1 months and its median OS was 11.5 months irrespective of patients’ PD-L1 status. Despite being well-tolerated, nivolumab led to grade 2 pneumonitis and grade 3 anemia in about 5% of the patients [16]. Consequently, monotherapy with anti-PD-1 monoclonal antibodies contributes to durable clinical outcomes with tolerable toxicity [17].

### 4.2. Breast Cancer

The high prevalence of breast cancer as well as the fact that this disease can still be diagnosed at a late stage or show minimal response to other treatments creates a need for the involvement of immunotherapeutic agents in this field, as well. As far as hormone receptor (ER and PR) negative and HER2-negative triple-negative breast cancer (TNBC) is concerned, the checkpoint inhibitor atezolizumab has been approved by the FDA for use with the chemotherapeutic agent nab-paclitaxel in patients with advanced TNBC (level of evidence I, A). This suggestion stands as long as PD-L1 stained, tumor-infiltrating immune cells of any intensity cover ≥1% of the tumor area. The TNT phase III clinical trial provided modest but statistically significant evidence favoring atezolizumab in terms of progression-free survival [18]. More specifically, the median PFS was 7.2 months in the patients with atezolizumab plus nab-paclitaxel, in comparison with 5.5 months of the group which received placebo plus nab-paclitaxel (HR: 0.80; 95% confidence interval [CI], 0.69 to 0.92; *p* = 0.002), whereas the mPFS was 7.5 months and 5.0 months, respectively among patients with PD-L1 positive tumors, (HR: 0.62; 95% CI, 0.49 to 0.78; *p* < 0.001). Furthermore, the median OS benefit in favor of the immunotherapy arm was approximately 10 months (25 months versus 15.5 months, HR, 0.62; 95% CI, 0.45 to 0.86) in case of PD-L1 positive malignancies, whereas it was estimated 3.7 months (21.3 months versus 17.6 months, HR, 0.84; 95% CI, 0.69 to 1.02; *p* = 0.08); among patients with PD-L1 negative tumors. Consequently, both groups have reaped the PFS benefit of immunotherapy irrespective of the PD-L1 status. However, noteworthy is the fact that no statistically significant benefit has been documented regarding overall survival [19]. Furthermore, the administration of pembrolizumab in tumors with mismatch-repair deficiency should be taken into account by attending oncologists (level of evidence I, C) via extrapolating the data of the aforementioned practice-changing clinical study that had been published in New England Journal of Medicine in 2015 [14].

### 4.3. Colorectal Cancer

Anti-PD-1 agents, pembrolizumab and nivolumab as well as the combination of nivolumab with ipilimumab are currently investigated in the context of colorectal cancer (CRC). Evidence for pembrolizumab derives from a phase 2 trial by Dung et al. in 2015. 41 patients with adenocarcinomas (32 of 41 with CRC) were treated with pembrolizumab for the treatment of tumors with and tumors without mismatch-repair deficiency (MMR-d) investigating the hypothesis that MMR-d tumors are more responsive to PD-1 blockade in comparison to mismatch repair proficient tumors [14]. The results were encouraging and hence pembrolizumab can be further tested to other cancers with mismatch-repair deficiency including those of the uterus, stomach, biliary tract, pancreas, ovary, prostate, and small intestine [19,20]. The reason why the aforementioned study is considered to be included in the recent milestones of oncologic research lies in the fact that it has managed to provide a license to an immune agent based on a molecular feature instead of abiding by the established tissue-specific approach [21]. Recently, a phase 3 study of pembrolizumab in microsatellite instability high advanced CRC was published, in which immunotherapy has been proven to be superior to chemotherapy regarding both mPFS (16.5 versus 8.2 months; HR, 0.60; 95% (CI), 0.45 to 0.80; *p* = 0.0002) and mOS after 1 year of follow-up (13.7 months versus 10.8 months). ORR was 43.8% of the patients in the immunotherapy arm in comparison with 33.1% in the chemotherapy arm. Last but not least, there was a threefold increase in the treatment-related toxicity grade 3 or higher when it comes to patients who received chemotherapy compared with those who received pembrolizumab (66% versus 22%) [22].

Nivolumab in a phase II trial (CheckMate 142) demonstrated activity in patients with microsatellite instability (MSI)-high or mismatch repair deficient metastatic colorectal cancer [23]. Following that, investigators conducted an international, multicenter, phase II trial to examine the potential impact of a combination treatment with nivolumab and the anti-CTLA-4 agent ipilimumab as a first-line treatment for the complete population of CheckMate. Patients received nivolumab (3 mg/kg every 3 weeks) plus ipilimumab (1 mg/kg every 3 weeks) for 4 doses followed by nivolumab (3 mg/kg every 2 weeks) until disease progression. Within 3.4 months, 49% of patients showed a response to treatment with 4% showing complete response; control of the disease was seen in 79% regardless of BRAF, KRAS mutations and PDL-1 status. 12-month overall survival was estimated to 85% whereas a grade 3 or 4 adverse event occurred in 32% of the patients [24]. In an updated analysis of the CheckMate 142 trial, the combined immunotherapy yielded a disease control rate of 84%, an objective response rate of 64% and a complete response rate of 9% [23].

### 4.4. Gynecological Malignancies

The extent of antitumor activity of immunotherapy in gynecological malignancies has yet to be established. Among these malignancies, besides cervical cancer, the inhibition of the PD-1/PD-L1 pathway may also be beneficial to women who have been diagnosed with ovarian cancer and vulvar cancer. There is a plethora of ongoing studies when it comes to the efficacy of immune checkpoint inhibitors (ICIs) against cervical cancer. Initial studies of pembrolizumab, nivolumab, atezolizumab and durvalumab either as single-agents at the metastatic disease or in combination with chemoradiotherapy at the locally advanced setting have yet to bear encouraging results regarding cervical cancer [23,24]. As far as ovarian cancer is concerned, the mono-immunotherapy approach has hitherto not been indicated, because of controversial data. Particularly, single-agent Abs against CTLA-4, PD-1 or PD-L1 revealed modest results in epithelial ovarian cancer with median response rates (RR) less than 15% and a control of disease rate less than 50% [25,26]. On the contrary, combinations of immunotherapy with chemotherapy, anti-angiogenic agents or poly (ADP-ribose) polymerase (PARP) inhibitors have shown promising results in terms of ORR and median PFS [27]. However, monotherapy with pembrolizumab in women with MSI-high tumors seems to be a reasonable therapeutic option irrespective of the tumor location as it has already been mentioned above [14]. The future aspects of immunotherapy in the management of gynecological cancer are likely to be illuminated by the highly anticipated results of the ongoing trials. 

### 4.5. Head and Neck Cancer

As far as head and neck cancer is concerned, pembrolizumab has received FDA approval in combination with platinum-based chemotherapy or as a single agent for patients with high PD-1 expression. The KEYNOTE-048 open-label phase III trial included 882 patients with inoperable recurrent or metastatic head and neck squamous cell carcinoma treated with single-agent pembrolizumab, chemotherapy plus pembrolizumab or a combination of targeted therapies (cetuximab) and chemotherapy (platinum, fluorouracil). Within 13 months of evaluation, the combination of pembrolizumab and chemotherapy demonstrated improved overall survival in patients expressing PD-1, and especially those expressing high-level PD-1 (according to PD-L1 combined positive score (CPS) stain gradient) in comparison to other regimens. More particularly, monotherapy with pembrolizumab yielded an OS benefit up to 4.2 months versus cetuximab with chemotherapy (in the CPS of 20 or more population: median 14.9 months vs. 10.7 months, ((HR) 0.61 (95% CI 0.45–0.83), *p* = 0.0007) and in the CPS of 1 or more population: 12.3 vs. 10.3, 0.78 (0.64–0.96), *p* = 0.0086). Moreover, the combination of pembrolizumab with chemotherapy led to OS benefit up to 3.7 months versus cetuximab with chemotherapy (in the total population: 13.0 months vs. 10.7 months, HR 0.77 (95% CI 0.63–0.93), *p* = 0.0034, in the CPS of 20 or more population: 14.7 vs. 11.0, 0.60 (0.45–0.82), *p* = 0.0004 and in the CPS of 1 or more population: (13.6 vs. 10.4, 0.65 (0.53–0.80), *p* < 0.0001). On the contrary, PFS benefit has not been observed in favor of immunotherapy in this study [28].

As far as metastatic nasopharyngeal cancer is concerned, to date, there is no available immunotherapy. However, several experimental approaches are spanning from adoptive immunotherapy to active immunotherapy. Adoptive immunotherapy is based on the direct activation of effector immune cells, while active immunotherapy is using Epstein–Barr virus (EBV) vaccination to stimulate tumor antigen recognition by the host immune system. Anti-PD-1 agents are also assessed in the context of metastatic nasopharyngeal carcinoma [29,30,31].

Active immunotherapy is being investigated with the form of vaccines. Three trials have been reported so far. Three phase II trials testing a vaccine based on dendritic cells. The first study recruited 16 patients with local recurrence or distal metastasis. More than 50% of them reported favorable outcomes. The second study also included 16 patients with stage II/III nasopharyngeal cancer. More than 9 patients reported favorable outcomes, however, 9 patients presented with delayed hypersensitivity later. The third trial focused on patients with extensive metastatic disease and reported poor outcomes. More potent vaccines administered at the early stage of the disease ought to be studied [29].

Checkpoint inhibitors are investigated in the KEYNOTE-028 study. 27 patients who have received treatment previously being diagnosed with, PD-L1 positive, metastatic or recurrent nasopharyngeal carcinoma were treated with pembrolizumab (10 mg/kg every 2 weeks for up to two years). 26% of the patients showed a partial response. Disease stabilization was achieved in 14 patients (52%) who were followed-up for a median of 20 months. The median progression-free survival reached 6.5 months, with progression-free survival 50 and 33% rates at 6 and 12 months, respectively [28,29].

Another phase II study included 44 patients, who were on nivolumab (3 mg/kg every two weeks) having previously received at least one dose of platinum-based chemotherapy for recurrent disease. With 12.5 months of a median follow-up, the objective response rate reached 20.5 percent. One patient showed a complete response and eight showed partial responses. The one-year progression-free rate reached 19% and the overall survival rate was 59% [32].

To date, two ongoing randomized trials have been acknowledged. They investigate pembrolizumab (KEYNOTE-122, NCT02611960) or a different anti-PD-1 antibody (NCT02605967), known as PDR001, in comparison to the indicated chemotherapy regimens for recurrent or metastatic nasopharyngeal cancer that progressed after platinum-based chemotherapy [31,32].

### 4.6. Hepatocellular Carcinoma

Immunotherapy for hepatocellular cancer (HCC) has emerged in recent years due to its predominance among liver cancers, its prevalence among cancers in general and the paucity of treatments [33,34,35]. In a global, open-label, phase 3 trial, patients with the combination of atezolizumab plus bevacizumab have been examined in comparison to sorafenib in the context of unresectable hepatocellular carcinoma. Totals of 336 and 165 patients, who had not previously received systemic treatment, were randomly assigned to the atezolizumab–bevacizumab and the sorafenib group. The combination of atezolizumab and bevacizumab was more effective in terms of overall survival at 12 months (67.2% vs. 54.6% with sorafenib) and median progression-free survival was 6.8 months vs. 4.3 months with sorafenib (HR for PD or death 0.59; 95% CI, 0.47 to 0.76; *p* < 0.001). Grade 3 or 4 adverse events, mainly hypertension, were reported in 56.5% of the atezolizumab–bevacizumab group and 55.1% of the sorafenib group. These findings suggest the combination of atezolizumab–bevacizumab as a first-line treatment for unresectable HCC [36].

Pembrolizumab has recently received accelerated access approval from the FDA as a second-line treatment after sorafenib based on an open-label, phase II KEYNOTE-224 trial including 104 patients were not responsive to sorafenib. Pembrolizumab was associated with an objective response of 17% with a complete response in one patient, partial response in 17 patients, and disease stabilization in 46 patients [37]. However, the comparison of pembrolizumab to placebo as a second-line HCC treatment in phase III CheckMate 459 and phase III Keynote-240 trials failed to meet its overall survival and progression-free survival goals [38]. Similar results have been demonstrated in the phase I/II trial CheckMate 040 of nivolumab, which included patients who had already received sorafenib, revealed an overall survival of 15.6 months [39]. However, so far phase III trials have also failed to deliver statistically significant results [38]. Moreover, it is worth mentioning that response rates of these immune checkpoint inhibitors do not exceed 25%, whereas grade 3/4 immune-related adverse events, such as rash, diarrhea, pruritus and increase of transaminases, have been documented [40].

Tremelimumab, a monoclonal anti-CTLA4 antibody was assessed in a phase II multi-center clinical trial including 20 patients with advanced HCC from hepatitis C viral etiology. The administration of tremelimumab at the dose of 15 mg/Kg intravenous (IV) every three months lead 18% of patients to partial response and 60% of patients of disease stabilization with progression-free survival of 6.48 months [41].

Testing a variety of agents and combinations of them in patients with HCC is of paramount importance, therefore, at present, numerous clinical trials are active. A striking example is the phase I trial of the combination of pembrolizumab and lenvatinib, which was well tolerated with promising anti-tumor activity in patients with unresectable HCC [42,43]. Furthermore, it is worth to mention that there are two ongoing phases 3 studies, the COSMIC-312 study and the HIMALAYA study, in which combination regimens are being evaluated. The former study refers to the combination of cabozantinib and atezolizumab versus sorafenib in patients with advanced HCC who receive their first-line of treatment and the latter regards the combination of tremelimumab, with an anti-PDL-1 immune agent, named durvalumab, which revealed tolerable toxicity and encouraging activity in patients with advanced HCC [36,40,41]. In addition, the combined treatment of nivolumab with ipilimumab led to significant response rates (30%) but also revealed increased toxicity compared to monotherapy with nivolumab [44]. Moreover, monotherapy with avelumab, which is an anti-PD-L1 antibody, as a second-line treatment and its combination with axitinib as a first-line treatment are currently being tested [43,44]. Finally, the experimental investigation of novel sophisticated treatments, such as adoptive cell therapy using T-cell engineering, cancer vaccines and oncolytic virus therapies form an evolving field, which promotes an unprecedented approach of the systematic management of advanced HCC. The modified poxvirus JX-594 is currently the prominent oncolytic virus of interest for HCC having conferred a dose-related survival benefit in a phase II dose-finding trial with 30 patients. A global, randomized, open-label, phase III trial is also assessing Pexa-Vec (JX-594—an oncolytic virus) providing to patients with two arms of vaccination with sorafenib versus sorafenib alone [44,45].

### 4.7. Lung Cancer 

Various forms of lung cancer have been a target for immunotherapy. Recent evidence or ongoing studies focus on resectable non-small lung cancer (NSCLC), extensive-stage small cell lung cancer, metastatic small cell lung cancer and refractory and relapsed small cell lung cancer [46,47,48].

Choosing the treatment of NSCLC lacking a driver mutation takes into consideration the level of PD-L1 expression, the extent of disease and pathology. Recently, two clinical trials concerning combinations of chemotherapy with immunotherapy constitute the hallmarks of the current therapeutic manipulations of metastatic lung cancer. Firstly, according to the KEYNOTE-407 clinical trial, the addition of pembrolizumab to chemotherapy with carboplatin plus nab-paclitaxel or paclitaxel contributed to significantly longer OS (15.9 versus 11.3 months, 95% [CI]) and PFS (6.4 versus 4.8 months, 95% [CI]) than chemotherapy alone regardless of the level of PD-L1 expression [49]. On the other hand, the IMpower150 clinical trial has revealed that the addition of atezolizumab to bevacizumab plus chemotherapy significantly improved OS (19.2 versus 14.7 months; hazard ratio for death, 0.78; 95% [CI], *p* = 0.02) and PFS (8.3 versus 6.8 months; hazard ratio for PD or death, 0.62; 95% [CI]) among patients with metastatic non squamous NSCLC, regardless of PD-L1 expression and EGFR or ALK genetic alteration status [50]. These advances have been integrated in the latest expert guidelines from The American Society of Clinical Oncology and Ontario Health, which have suggested pembrolizumab as monotherapy in cancers with PD-L1 expression ≥50 percent. Rapidly progressing or very extensive disease may be treated with a combination of platinum-based chemotherapy and pembrolizumab. The same suggestion applies to cancers with PD-L1 expression <50 percent [51]. The addition of anti-PD-1 or PD-L1 treatment before or concurrently with osimertinib in patients with epidermal growth factor receptor (EGFR) mutations has been halted due to the increased risk of pulmonary toxicity [52].

Anti-PD-1 and anti-CTLA4 based immunotherapies are investigated as adjuvant or neo-adjuvant treatments for resectable NSCLC. More specifically, adjuvant treatment aims to the regression of micrometastatic disease while neoadjuvant treatment aims to “downstage” primary tumors or decrease the morbid effects of surgery and enable a subsequent analysis of the treated tumor. Phase II trials currently exploring immune checkpoint inhibitors include the NEOMUN (NCT03197467) and NADIM (NCT03081689) studies, as well as the platform NeoCOAST (NCT03794544) trial [51,52,53].

NSCLC metastasizing to the brain can also be a target of immunotherapy. Experience in this field is limited, given that the existing insights derive from the use of anti-PD-1 agents to resectable NSCLC with significant PD-1/PD-L1 presence. Old evidence supported PD-1/PD-L1 based immunotherapy as monotherapy with promising objective intracranial responses in up to 30% of patients with NSCLC related-brain metastases. A retrospective study of 255 patients suggests durable intracranial outcomes that can be accompanied by favorable extracranial responses in 13% of patients [54,55]. This concept has been lately revisited with recent clinical trials/expanded access programs to nivolumab or atezolizumab reporting favorable outcomes in up to 39% of patients [54,56].

Concerning the initial management of extensive-stage SCLC, immunotherapy can be combined with chemotherapy according to the latest evidence [49]. Anti PD-L1 agents, atezolizumab and durvalumab have increased survival, in combination with a platinum agent and etoposide as induction and maintenance treatments. Existing evidence from cross-trial comparisons suggests a similar level of efficacy and toxicities between durvalumab and atezolizumab in combination with chemotherapy. Hence, the exact treatment can be decided according to the preference of the provider, availability, experience and insurance coverage [54].

When it comes to refractory and relapsed small cell lung cancer, immunotherapy is considered as a first choice second-line treatment. Nivolumab, alone or in combination with ipilimumab is evidenced for most patients with extensive SCLC after progression on initial chemotherapy, provided that: (1) they did not also receive immunotherapy in the frontline setting; (2) the relapse did not occur within 6-months—in that case, chemotherapy is preferred. Pembrolizumab has also received approval by the FDA for patients with metastatic SCLC who have experienced progression on or after platinum-based chemotherapy and at least one other line of therapy. Ongoing clinical trials investigate whether pembrolizumab stands as a monotherapy for patients who experienced adverse events with nivolumab and ipilimumab [57,58].

### 4.8. Lymphoma

Chemoimmunotherapy has been established as the cornerstone of the management of non-Hodgkin’s lymphoma (NHL) for decades. More specifically, it has been documented by a majority of studies and meta-analyses that the combination of conventional chemotherapy with rituximab, which is an anti-CD20 agent, contributed to superior RR and OS [59]. Consequently, the concurrent administration of many different chemotherapy regimens and anti-CD20 monoclonal antibodies, such as rituximab and lately obinutuzumab, has constituted the “standard of care” approach regarding the treatment of relapsed or refractory (NHL) [60].

As far as classic Hodgkin’s lymphoma (HL) is concerned, there is a very alluring strategy that entails the delivery of chemotherapy to CD30-expressing cells, such as the Reed Sternberg (RS) cells of HL embracing the assistance of the antibody-drug conjugate, brentuximab vedotin [61]. When it comes to anti-PD-1 monoclonal antibodies, nivolumab and pembrolizumab (KEYNOTE-087) have demonstrated some efficacy concerning HL, but their durability of response remains in question. Last but not least, pneumonitis, colitis, hepatitis, thyroid dysfunction, nephritis and renal dysfunction were included in their adverse events [62,63]. Finally, evolving immunotherapy approaches include adoptive immunotherapy with cytotoxic T-lymphocytes which are specific for RS cells or EBV latent antigens and CAR T-cells that bear chimeric antigen receptors (CARs) against malignant cells, but their efficacy has yet to be determined [64,65].

### 4.9. Melanoma

Cutaneous melanoma as well as advanced melanoma can be treated with immunotherapy. Nivolumab was approved by the US Food and Drug Administration (FDA) in December 2017 for adjuvant treatment of patients who had undergone definitive resection of cutaneous melanoma and had metastatic lymph node involvement, and for patients with stage IV disease who had undergone definitive resection of all sites of disease. Following surgical resection of cutaneous melanoma, nivolumab showed superiority over ipilimumab [66]. Pembrolizumab has also prolonged progression-free survival in patients with cutaneous melanoma regardless of the presence of PD-1. However, pembrolizumab has been associated with more severe adverse effects, including myositis [65,66].

Checkpoint inhibitors, such as nivolumab, ipilimumab and pembrolizumab have recently been approved for the treatment of advanced melanoma. Monotherapy with ipilimumab tends to be replaced by nivolumab and pembrolizumab due to its toxicity with an exception for BRAF wild type melanoma, which seems to respond better to ipilimumab. Nivolumab has been introduced to the treatment of advanced melanoma in 2015 and has been further supported by trials published in 2019 and its dosage was later adjusted based on more recent evidence [64,67,68,69]. Furthermore, the combination of nivolumab and ipilimumab for previously untreated patients with advanced melanoma seems to be a highly promising choice with a favorable outcome, given that the 5-year OS of the combination group has been demonstrated to be significantly higher (52%) in comparison with the groups that received monotherapy with either nivolumab (44%) or ipilimumab (26%), without some deterioration of health-related quality of life can be substantiated [70]. An ongoing clinical trial in Germany, IMMUNED, is currently reassessing the combination of nivolumab and ipilimumab in comparison to nivolumab [71]. As far as pembrolizumab is concerned, it has greatly been approved grace to the KEYNOTE 054 phase III clinical trial in Europe, in which it showed supremacy over placebo concerning high-risk stage III melanoma. Specifically, as far as the total population is concerned, the recurrence-free survival was longer regarding the pembrolizumab group in comparison with the placebo group—75.4% (95% CI, 71.3 to 78.9) versus 61.0% (95% CI, 56.5 to 65.1); HR for recurrence or death, 0.57; 98.4% CI, 0.43 to 0.74; (*p* < 0.001) [68]. Furthermore, 5-year survival outcomes for 655 patients with advanced melanoma treated with pembrolizumab were evaluated in the KEYNOTE-001 study. According to this study, the estimated 5-year OS was 34% in the total population and 41% in the treatment-naïve population, whereas mOS was 23.8 months (95% CI, 20.2–30.4) and 38.6 months (95% CI, 27.2–not reached), respectively [72].

### 4.10. Neuroendocrine Tumors

As far as neuroendocrine tumors (NETs) of the gastrointestinal tract are concerned immune checkpoint inhibitors immunotherapy has attracted the attention for the treatment of patients with well-differentiated NETs. Limited data suggest that anti- PD-1 antibodies have minimal activity as single-agent therapy. Spartalizumab (PDR001), which was evaluated in a multicenter phase II trial with including 116 patients with various NETs reported a radiographic response rate of 7.4% for well-differentiated NETs and 0% for gastrointestinal neuroendocrine tumors (GI NET). The stable disease rate in almost 2/3 of patients with both well-differentiated NET and GI NET, was quite promising though [73,74].

The KEYNOTE-028 study assessed pembrolizumab in the context of well-differentiated PD-1 positive gastrointestinal and thoracic NETs. Objective responses were limited to few patients with non-pancreatic NETs and the stable disease rate among all the patients was 60% [75]. Similar outcomes concerning pembrolizumab were seen in the phase II KEYNOTE-158 trial [76]. Ongoing clinical trials focus on the combination of immunotherapy with targeted therapies [77].

### 4.11. Castration-Resistant Prostate Cancer

The immunotherapy approach for castration-resistant prostate cancer is twofold. It consists of anti-PD-1 agents and a vaccine. KEYNOTE 028 and KEYNOTE 199 phase II trial suggest that pembrolizumab can result in durable responses in castration-resistant prostate cancer as long as the tumors express PD-L1 in ≥1% of tumor or stromal cells. Partial responses accounted for 17% while stable disease accounted for up to 35%. The median duration of response was 13.5 months [14,72].

KEYNOTE-199 phase II study studied the effect of pembrolizumab in 258 men with docetaxel-refractory metastatic prostate cancer suggesting higher efficacy of pembrolizumab in patients with PD-L-1 overexpression or disease spread to the bones [78].

Recently, the vaccine sipuleucel-T was studied in three randomized trials. In the first two trials the primary endpoint was progression-free survival, and overall survival was a planned secondary endpoint. A subsequent, larger phase III trial was designed with overall survival as the primary endpoint. Patients were allocated randomly at a 2:1 ratio against placebo, and the primary outcomes appear to be encouraging enough in patients with advanced disease, including local and peritoneal metastases [79].

### 4.12. Renal Cell Carcinoma

Immunotherapy seems to have more applications in the advanced RCC. When it comes to localized RCC, immunotherapeutic agents have failed to show statistically significant outcomes in phase II clinical trials, while several vaccines have also been of controversial efficacy [76,77].

Advanced clear cell renal cancer can be initially treated with immunotherapy, targeted therapy, or a combination of both including nivolumab and pembrolizumab, avelumab and atezolizumab, ipilimumab and/or VEGF inhibitors such as axitinib, sunitinib, pazopanib, and bevacizumab. Most recent evidence suggests combinations of nivolumab and ipilimumab or pembrolizumab and axitinib for symptomatic patients with a significant burden of disease. The superiority of these agents over sunitinib as first-line treatments has been supported by phase III clinical trials [78,79,80]. The supremacy of either combination is still debatable because of the lack of data. Existing evidence from cross-trial comparisons suggests a better OS hazard ratio with pembrolizumab plus axitinib, but potentially more durable responses with nivolumab plus ipilimumab. More specifically, the mOS was not reached with nivolumab plus ipilimumab, whereas the mOS of patients who received sunitinib was 26.0 months (HR for death, 0.63; *p* < 0.001). As far as the combination of pembrolizumab and axitinib is concerned, mOS was not also reached vs. 35.7 months (95% CI 33.3—not reached) with sunitinib; ((HR) 0.68 (95% CI 0.55–0.85), *p* = 0.0003) and mPFS was 15.4 months (12.7–18.9) vs. 11.1 months (9.1–12.5); ((HR) 0.71 (0.60–0.84), *p* < 0.0001) respectively. [81,82,83] Other scholars revisit immunotherapy-targeted combination therapy with avelumab plus axitinib or single-agent pembrolizumab, as documented in 2012 [82]. The combination of axitinib plus avelumab improved the progression-free survival, although other parameters are yet to be defined [83].

### 4.13. Soft Tissue Sarcomas (STS)

As far as advanced STS are concerned, immunotherapy has only been indicated in case of MSI high tumors. Monotherapy with pembrolizumab, the combination of pembrolizumab and axitinib, which is an anti-angiogenic agent, as well as nivolumab with or without ipilimumab have been studied, but they have yet to yield promising results [84,85]. To sum up, it all boils down to the fact that immunotherapy remains an exploratory domain in the management of STS and it is not included in the “standard of care” practice [86].

### 4.14. Urothelial Cancer

Immunotherapy can be applied to metastatic or advanced urothelial carcinoma. Recent evidence supports the administration of anti-PD-1 and anti-PD-L1 to patients who have progressed during or after platinum-based chemotherapy. According to the KEYNOTE-045 trial, pembrolizumab showed an OS benefit up to 2.9 months as second-line therapy for advanced urothelial carcinoma as compared to the chemotherapy group ((HR) for death, 0.73; 95% CI, 0.59 to 0.91; *p* = 0.002) [87]. Besides pembrolizumab, FDA approved ICIs include nivolumab, atezolizumab, avelumab, and durvalumab. Although the efficacy of these agents has been documented, it seems that the expression of PD-1 is not reliable when it comes to defining immunotherapy for urothelial carcinoma. Hence, current research focuses on other potential biomarkers capable of defining the optimal agent [88].

Moreover, safety concerns regarding patients with low levels of PD-L1 expression have been raised by two randomized phase III trials, KEYNOTE-361 and IMvigor130. Hence, atezolizumab and pembrolizumab are now indicated for cisplatin-ineligible patients with advanced bladder cancer who have low PD-L1 expression or are ineligible for any platinum-containing chemotherapy [89,90].

## 5. Toxicity

Cancer immunotherapy—either active or passive—manipulates the immune system inducing durable effects on cellular, tissue and organic levels. These effects are primarily toxic for tumors but patients may also suffer their consequences; cancer immunotherapies can lead to unique toxicity profiles distinct from the toxicities of other cancer therapies, depending on their mechanism of action [91].

Immune checkpoint inhibitors and CAR-T among others have revolutionized the treatment of multiple malignancies, including but not limited to melanoma, RCC, lung cancer (both small cell and non–small cell), head and neck squamous cell carcinoma, gastric cancer, ovarian cancer, Hodgkin lymphoma, and tumors with DNA mismatch repair defects have been correlated with various inflammatory side effects, also known as immune-related adverse events (irAEs). Given that indications for ICIs therapy have been expanded and multiple clinical trials for both solid and hematologic malignancies have been completed, several aspects of ICIs toxicity ought to be considered. 

The principal recorded toxicity include colitis/diarrhea, hypophysitis, thyroid dysfunction, dermatological lesions and hepatitis [92]. From a pathophysiologic aspect, IL-17, a cytokine associated with bowel inflammation, has been found elevated in patients with melanoma treated with neoadjuvant ipilimumab, who presented with grade 3 diarrhea or colitis [93].

As far as hypophysitis is concerned, patients with ipilimumab-induced hypophysitis may develop antibodies against thyroid-stimulating hormone (TSH) secreting cells and some of them may also develop antibodies against follicle-stimulating hormone-secreting or adrenocorticotropic hormone (ACTH) secreting cells [94]. Defects in the associated hormone axis have been attributed to the presence of these antibodies. Another hypothesis involved the physiological CTLA-4 expression pituitary tissue, as a target of ipilimumab-induced antiCTLA-4. Relevant findings derive from an autopsy study of 6 patients treated with CTLA-4 blockade. CTLA-4 was expressed in the pituitary of all the patients and the higher the level of expression, the higher the severity of hypophysitis was [91].

Thyroid dysfunction, predominantly hypothyroidism, has been associated with PD-1 blockade when a study of patients with advanced non small cell lung cancer (NSCLC) treated with pembrolizumab reported the presence of antithyroid antibodies in 80% of patients who developed hypothyroidism compared with 8% of patients who did not. According to these findings, PD-1 toxicity seems to be mediated by humoral immunity but further investigation ought to be conducted [45].

Pneumonitis is the most common and life-threatening pulmonary toxicity of ICI therapy. A meta-analysis of fatal side effects of ICI reported that about one third of antiPD-1 and antiPD-L1 related fatalities occurred because of pneumonitis. Dual checkpoint inhibition and PD-1 monotherapy are more likely to cause pneumonitis in comparison to CTLA-4 monotherapy [95]. A large retrospective study with 915 patients treated for multiple tumor types with antiPD-1 and antiPD-L1 monotherapy or combination therapy reported an overall 5% incidence of pneumonitis, with 1% to 2% grade 3 and 4 pneumonitis. According to the same study, pneumonitis in the context of PD-1 immunotherapy is more common and more severe in patients suffering from NSCLC compared with those who have melanoma [96].

Renal toxicity irAEs are rare. According to a review of published phase 2 and 3 trials, their incidence does not exceed 2% of patients receiving ICI monotherapy and 5% of patients receiving combination therapy [97]. More recent studies have reported a higher incidence of acute kidney injury, especially in patients treated with ICI. Apart from immunological pathogenesis, these findings could reflect either checkpoint inhibitor toxicity or other well-known causes of acute kidney injury, such dehydration and other nephrotoxic medications. The presentation of renal irAEs spans from worsening hypertension, electrolyte imbalance and altered urinary output to rising creatinine [98].

Cytokine release syndrome (CRS) has been reported after the infusion of CAR-T CD19 therapy. Its mechanism has been attributed to T-cell activation following the engagement of CAR-T cells with their targets. The initial presentation consists of constitutional symptoms such as malaise, myalgias, fatigue, and rash, with fever being required for the diagnosis of CRS [99]. Although CRS may be self-limited or may regress with supportive care, it can become life-threatening, with capillary leak leading to peripheral and pulmonary edema, hypotension, multiorgan failure, and circulatory collapse. It usually occurs 1 to 14 days after infusion. Patients with severe CRS manifest hepatosplenomegaly, hepatic dysfunction, hyperferritinemia, hypofibrinogenemia, and coagulopathy [100].

## 6. Discussion 

During the last 10 years, the therapeutic applications of immunotherapy have been explored to a great extent, providing healthcare professionals and researchers with approved and promising treatments. Immunotherapeutic agents have shown their potential as only-agents or combination either with one another or with chemotherapy, targeted therapy and invasive procedures. Attention has also been paid to vaccines and their therapeutic applications, a viewpoint that may change the perception of vaccines in the future.

An attempt to predict the future of cancer immunotherapy requires an inclusive viewpoint spanning from promising immunotherapy regimens to health policy in terms of access to medicines and sustainability. When it comes to promising immunotherapy regiments, some studies focus on therapeutic targets applicable to several malignancies, others follow a malignancy-specific approach and others address advanced malignancies, that have either remained or failed to be treated. There is a wealth of concepts and studies that are either in preclinical stages or have received approval for Phase 1 clinical trials regarding the innate immunity targeting immunotherapy [101].

The innate immune system provides a wealth of therapeutic targets. Overall, it is considered that several malignancies tend to escape immune responses by tackling innate immunity and subsequently antigen-specific adaptive immunity. Toll-like receptors (TLRs) have already been targeted in bladder cancer (BCG immunotherapy) or breast cancer (Imiquimod). A novel concept suggests that TLRs exist on tumor cells and can work as tumor markers and targets triggering cytotoxic activity and immune effector cells to extinguish cancer cells [102]. This has gained significance taking into account recent evidence concerning the involvement of TLRs in metastasis [103]. Moreover, preliminary data suggest that combination therapy with TLR ligands and conventional radiation/chemotherapy can have a higher growth-inhibitory effect compared to monotherapy patterns. Decreasing the clinical dosage of chemotherapy and the associated side effects is a considerable asset of this approach [104].

RIG-I like receptors (RLRs) are a family of DExD/H box RNA helicases playing an important role in antiviral immunity. The activation of RLRs induces an immunological torrent resulting in the release of interferon (INF) and the apoptosis of the virus-host cell. Mimicking this mechanism offers hope for novel cancer immunotherapy agents. The principal target is the 5′ ppp-SiRNA (triphosphate small-interfering RNA) which induces TGF-b production enhancing immunosuppression. This pattern has been particularly observed in pancreatic cancer and melanoma [105]. A viral RLRs trigger, the hemagglutinating virus of Japan envelope (HVJ-E) vector, has been tested in phase I clinical trials for melanoma, malignant pleural mesothelioma, and castration-resistant prostate cancer (CRPC). Conceptual barriers such as the optimal ligands targets for delivery to the tumor and practical barriers such as the optimal combination partners (chemotherapy, radiation, active, passive immunotherapy) are yet to be addressed [106].

As far as CRC is concerned tackling molecular targets within the tumor immune microenvironment is a key concern for future treatments. CKD-516, the vascular disrupting agents, invokes Rho signaling to activate dendritic cells and to exhibit immune-modulatory effects. Synergistic action of CKD-516 combination and anti-PD-1 mAbs have given promising enough preclinical data for the SMAD-4 deficient MSS-CRC to proceed with the clinical testing of CKD-516/anti-PD1 combination in CRC patients with MSS tumors [107]. Targeting hematopoietic progenitor kinase 1 (HPK-1] seems to be promising, too. This approach is twofold. Firstly, HPK-1 is expressed on hematopoietic cells and but not solely on a specific type of cancer cells. As a result of this, it is considered a promising target to revert immune escape in a wide range of tumors including CRC. The second point is that HPK-1 inhibition demonstrated a lower incidence of systemic effects when compared to CTL-A4 inhibition. This observation supports the safety of approaches targeting HPK-1 [108]. Last but not least, the modulation of branched chain amino acid transaminase 1 (BCAT1) has been shown to induce metabolic reprograming of CD8+ T-cells and augmented activity of anti-PD-1 mAbs in preclinical studies. Both HPK-1 and BCAT1 modulatory approaches need to be assessed in the frame of clinical trials [20].

As far as advanced malignancies are concerned, inducing a T-cells and natural killer (NK) cells mediated immune response has recently been brought under investigation. A Phase 1a, open-label, dose-escalation study evaluating the safety, tolerability, and initial efficacy of recombinant human anti-T-cell immunoreceptor with Ig and ITIM domains (TIGIT) monoclonal antibody injection (IBI939) in subjects with advanced malignant tumors was initiated in March 2020, in China. IBI939 can directly bind to TIGIT, disturb the interaction between CD155 and TIGIT, relieve the inhibition and depletion of T cells and NK cells and enhance the anti-tumor immune response of T cells and NK cells [109].

## 7. Access to Medicines

Access to medicines is a major key aspect of novel anticancer immunotherapy. No matter how innovative or effective the current or future treatments may be, they will have no impact if they are not widely accessible.

Access to immunotherapy can be approached from two angles. The first one pertains to licensed and commercially available immunotherapies. In this case, the main challenge is financial. According to the “Let’s talk access” White Paper of the Association of European Cancer Leagues, in 2018 patients with metastatic melanoma in Eastern Europe had limited access to immunotherapy [110,111]. The same problem applies to conventional anticancer chemotherapy given that numerous patients across Europe experienced hardships with getting free and timely access to chemotherapy in the same year. The cause of this deficit was attributed to healthcare systems finance [106,107].

The second angle is related to compassionate access to unauthorized medicines. Any novel and evolving immunotherapy can be considered as an unauthorized medicine eligible for compassionate/palliative administration to patients who have failed continually to respond to the indicated licensed treatments [112,113]. Compassionate use is widely possible in the context of clinical trials, where eligible patients consent to explore a further chance for survival and quality of life in exchange of validating or debunking the efficacy of a new agent [108,109]. Clinical trials are conducted in tertiary university hospitals and there is no homogeneous recruitment procedure. It has been reported that most of the patients are unaware of what a clinical trial consists of and what chances compassionate access to novel medicines may give to them [97].

## 8. Legislative Issues

Back in 2014, the European Union (EU) Regulation for the European Medicine Agency (EMA) laid the fundaments of an open database where results from clinical trials will be openly distributed to investigators across the EU [114]. In terms of science, this process accelerates original research and meta-research by providing novel concepts and raw data respectively. In terms of health policy, this ensures transparency given that data are publicly available and claims of fabrication can be repelled on the grounds of evidence [110,111].

Broadening this policy to anticancer immunotherapy would be of paramount importance. Apart from growing data from ongoing clinical studies, researchers and clinicians would be able to access data from the use of licensed agents enhancing the reproducibility of the initial results that led to their license and perhaps the spectrum of their clinical applications. Data from the compassionate use of such agents would also be of crucial significance in accelerating the licensing of new agents or repurposing of existing ones [113,115].

In any case, compliance with the confidentiality standards as described by the General Data Protection Regulation (GDPR) within the EU and by relevant lawmaking elsewhere would be essential [116].

## 9. Anticancer Immunotherapy in the Frame of Sustainability

Sustainability is a vague concept and a challenge that contemporary research and healthcare faces. A wide accepted context of sustainability is the one described by the United Nations Sustainable Development Goals (UN SDGs). Anticancer immunotherapy apart from clinical practice and basic or translational research has also close ties with policymaking and finance [90,91].

Advances in immunotherapy serve UN SDG 3, the goal dedicated to health and wellbeing. At the same time, taking into account the chain of developing or repurposing a treatment from the lab bench to clinical trials and eventually to licensing and clinical practice, we understand that immunotherapy is aligned to UN SDGs 4, 9 and 10 (namely, quality education, innovation and reduced inequalities) [117,118]. Immunotherapy has been a synonym of innovation so far. Its development has already and may further spearhead the discussion about the involvement of industry and startups in healthcare. Quality education is also prompted by anticancer immunotherapy given that it is a matter of current postgraduate and future undergraduate medical education. Reduced inequalities apply both to healthcare professionals and patients [117]. Making immunotherapy globally accessible enhances reduced inequalities for cancer patients while training physicians and researchers worldwide in such standards promotes equal chances and opportunities in education and practice [113,114].

## 10. Conclusions

Regardless of the problems that have already appeared or will appear in the future, resuscitation continues to be one of the greatest achievements of our time. This is confirmed by the awarding of the Nobel Prize in Medicine and Physiology in 2018 to the two immunologists, the American James P. Allison and the Japanese Tasuku Honjo, for their innovative approach to the treatment of cancer. Immunotherapy is now a safe treatment option against cancer and many patients are already enjoying its benefits.

## Figures and Tables

**Table 1:** An overview of Immune Checkpoint Inhibitors, CTLA-4, Cytotoxic T-lymphocyte associated protein 4, PD-1, Programmed cell death protein 1, PD-L-1, Programmed death-ligand 1.

Drugs	Type	Mechanism of action	History	Approval
Ipilimumab	Anti-CTLA-4	Regulates T-cell activation and enhances immune responses with a focus on antitumor immunity	CTLA-4 was first detected in 1987. Its negative effect on T-cell activation was demonstrated in 1995	2011
Nivolumab, Tremelimumab	Anti-PD-1	Inhibition of PD-1, a protein downregulating the immune response and its antitumor effect	During 1990’s knockout mice for PD-1 were found vulnerable to autoimmune diseases	2014
Pembrolizumab, Atezolizumab, Durvalumab, Avelumab	Anti-PD-L-1	Immune system suppression	Was detected as a ligand to PD-1 with immune regulatory properties	2014, 2016, 2017 and 2017 respectively
